# Safety, effectiveness and hesitancy of COVID-19 vaccination in children: A cross-sectional study in Pakistan

**DOI:** 10.3389/fpubh.2022.1084017

**Published:** 2023-01-17

**Authors:** Zaufishan Zaufishan, Muhammad Usman, Khandah Fishan Mumtaz, Rabiea Bilal, Alina Arshad, Humaira Majeed Khan

**Affiliations:** ^1^Faculty of Pharmaceutical and Allied Health Sciences, Institute of Pharmacy, Lahore College for Women University, Lahore, Pakistan; ^2^Institute of Pharmaceutical Sciences, University of Veterinary and Animal Sciences, Lahore, Pakistan; ^3^Department of Pediatrics, Continental Medical College/ Hayat Memorial Hospital, Lahore, Pakistan; ^4^CMH Lahore Medical College and IOD, NUMS, Lahore, Pakistan

**Keywords:** COVID-19, children, safety, Pakistan, vaccine

## Abstract

**Background:**

The elevated risk of serious complications like myocarditis and pericarditis after COVID-19 vaccination, especially in adolescent has been reported in some instances that need to be tested in regional populations and different ethnicity groups. The purpose of the study was to evaluate the side effects, hesitancy, and effectiveness outcomes following COVID-19 vaccination among children in Pakistan.

**Methods:**

The study was planned using a cross-sectional design and data from Children and Adolescents (CA) was collected through a convenient sampling method using a validated questionnaire between February to July 2022. A total of 1,108 CA between the age of 12–18 years who received one or two doses of vaccine were selected and data were collected through direct interviews with respondents.

**Results:**

The results showed that among 99.8% of respondents who received the Pfizer COVID-19 vaccine, 72.3% of respondents were partially vaccinated (with one dose) while 27.7% were fully vaccinated (with two doses). COVID vaccination regime had a favorable safety profile in children as compared to adults. Vaccine hesitancy in children was reported to be 52.4% and the most common reasons for hesitance were the assumption that the vaccine is not safe (23.7%), the vaccine is not required (19.6%) and the vaccine is not effective (10.4%). The reported side effects were mainly mild (88.5%) followed by moderate (10.6%) and only 0.8% were of severe intensity. Post-vaccination local side effects of mild intensity were common with an onset of an average of 24 h (68%) and a duration of 2–3 days (60.6%). The reported side effects were significantly associated with gender (*p* = 0.00) while age had no significant effect on the occurrence of side effects. Overall, the vaccine was well tolerated by children and adolescents and was effective in preventing the reoccurrence of COVID-19 infection in 99.9% of participants.

**Conclusion:**

COVID-19 vaccine by Pfizer approved by the FDA for use in CA 12–18 years of age was well tolerated with a good safety profile and no serious adverse drug reactions were reported. The vaccine side effects were mild (88.5%) and lasted for an average of 2–3 days only (60.4%). The vaccine was effective in safeguarding Children against COVID-19 infection.

## Introduction

The coronavirus disease, denoted as COVID-19, has surfaced as one of the most devastating health problems and crises in the history of the last 100 years and was declared a global pandemic by WHO in March 2020. It originated in China and spread around the globe to 114 countries within the first three months and created havoc. The public health crisis was bigger than any other phenomenon in living memory (1). COVID-19 is an infectious disease prompted by a new virus Severe Acute Respiratory Syndrome Coronavirus 2 (SARS-CoV-2) which is highly contagious with rapid transferability among humans. WHO officially declared on 28 September 2022, a total of 642+ million (642,379,243) confirmed COVID-19 cases with 6,624,118 deaths as on 7th December 2022. Pakistan announced its first case of COVID-19 on 26 February 2020, escalating to its first peak in July 2020 with 1,572,410 confirmed cases with 30,612 deaths in Pakistan (WHO COVID Dashboard, https://COVID19.who.int/).

The devastation COVID-19 caused was across all age groups. CA contributed to 13% of the total confirmed COVID-19 cases in the US (2). The severity index of the disease, signs, and symptoms, and clinical presentation of COVID-19 in children varies from adults (3). COVID-19 cases in children are majorly asymptomatic (4) while symptomatic cases are usually of moderate to mild intensity with a low risk of hospitalization (5). Children having less severity of COVID-19 symptoms may be attributed to a decrease in ACE2 receptors which are primary target receptors for the virus with age (6). ACE2 has lung protective effects by reducing inflammation and pulmonary capillary leak. Also, due to frequent viral infections and live virus vaccines children have a better innate immune response as trained immunity leads to good early control of infection. Also, the regeneration capacity of alveolar epithelium in children is good and may also contribute to a speedy recovery. Children generally have fewer risk factors such as chronic illnesses, comorbidities, smoking, and obesity but young children with preexisting medical conditions could be at higher risk of COVID-19 (3).

With the increasing lethality of COVID-19, vaccination against SARS-CoV-2 was considered the best strategy to reduce infection rates, morbidity, and mortality in the overall population among all age groups (7). Moving forward, COVID-19 was declared a worldwide pandemic by WHO in March 2020 and less than a year later around 200 vaccines were rigorously been tested in preclinical trials by December 2020 (8). Some were tested and granted authorization for emergency use by FDA and the WHO for rapid vaccine rotation in countries around the globe. The world has been desperate for a cure as millions suffered from COVID-19 and its consequences (9). The candidate vaccines for clinical evaluation included viral vector vaccines, inactivated viruses, live attenuated viruses, and DNA and protein subunit vaccines. Some vaccine contenders across the world were granted authorization for emergency use by FDA and WHO with speedy vaccine rollout plans (10).

WHO and other international authorities in their efforts to ensure equitable access to safe and effective vaccines have successfully given 12,998,974,878 vaccine doses to populations across different countries (WHO Coronavirus Dashboard 28 September 2022).

Pfizer vaccine in a two-dose regimen imparts 95% protection against Coronavirus disease in more than 16 years of age persons. Comparable vaccine effectiveness of 90–100% was observed in population subgroups defined by age, ethnicity, gender, baseline BMI, and comorbidities (11). The clinical trials of the Pfizer vaccine in 12–15-year-old children had a favorable safety profile and were extremely effective against COVID-19. Also, the initial testing in children proved that it is effective in reducing the risk of infection in 5–15-year-old children, with only mild and short-term side effects mild (12).

Pfizer vaccine against COVID-19 resulted in the mild presentation of disease with less severe symptoms while the vaccination was majorly well tolerated by children and adolescents. Also, it was scientifically established that vaccine protection varies according to SARS CoV-2 variant type, i.e., lower protection for Omicron than Delta variant (5). Vaccination against COVID-19 in children had also a varied response in different age groups as children 5–11 years of age showed lower effectiveness in preventing SARS-CoV-2 infection than in children 12 years and above.

However, there was an elevated risk of serious complications like myocarditis and pericarditis after COVID-19 vaccination, especially in adolescent males in some instances that need to be tested in regional populations and different ethnicity groups (13). The approximation of vaccination safety and effectiveness in real-world settings may differ largely from trial data owing to factors like prior infection-induced immunity, herd immunity, the emergence of new variants, and evolving vaccination policies.

COVID-19 vaccination was first initiated on 3 February 2021 in Pakistan. Phasing out the vaccination plans for the masses, the priority was first given to frontline health workers, senior citizens, and people at high risk with comorbid health conditions. Vaccination was opened for 15 years plus ages from 13th September 2021 while it was further extended to those above 12 years of age from 28th September 2021 (14). Statistics of the number of children who received COVID-19 vaccination to date, its safety, and side effect profile in children in Pakistan are majorly unavailable from the official websites of the Ministry of Health and Ministry of Health Services Regulation and Coordination, Pakistan. Hence, we aim to validate the safety and effectiveness of COVID-19 vaccination in children from 12 to 18 years in Pakistan.

## Materials and methods

### Study design

This was a cross-sectional study designed to analyze the data on COVID-19 Vaccination in children and adolescents (12–18 years) in Pakistan. The data was collected between February to July 2022 through a validated questionnaire. A pilot study was conducted to validate the questionnaire. Data from most respondents was collected within 1–3 months of vaccination to minimize the limitations like recall bias. The inclusion criteria of participants were (a) healthy volunteer children who received COVID-19 vaccination in the last 2 years, (b) children who have received only one dose, both doses and booster doses of COVID-19 vaccination, and (c) All children from 12 to 18 years of age who received COVID-19 vaccination. The exclusion criteria were (a) Children who were not vaccinated for COVID-19. (b) Children with known allergies to any ingredient of the vaccine. (c) Children with any acute illness requiring emergency hospitalization in the last 1 month and (d) children with co-morbid conditions requiring active management plans like cancer chemotherapy. The ethical approval of the study was taken from the Office of Research Innovation and Commercialization (ORIC), Lahore College for Women University (LCWU), Lahore. Data was collected through interviews with the informed consent of participants, parents/ guardians, and teachers. Participants were identified with unique IDs for a diverse and representative sample. A convenient sampling technique was adopted for data collection. The data was collected from specific age groups of children from vaccination centers, hospitals, colleges, and schools.

## Study plan

**Figure 1:**
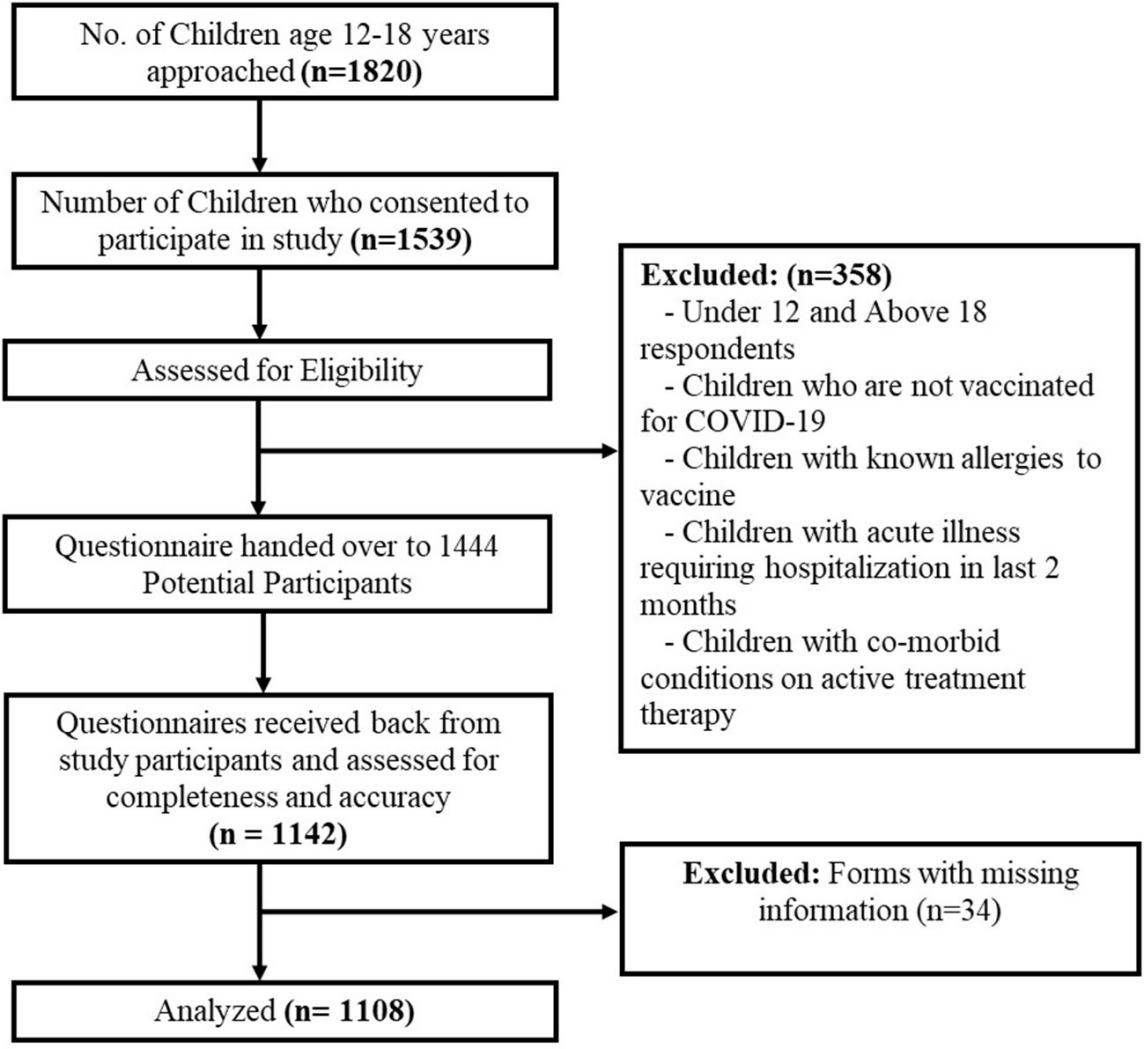
Strobe flowchart of response rate of study participants.

The study was planned and executed in a phased manner and included the development and validation of the study instrument in the pilot phase followed by the data collection and analysis phase. The flow chart for data collection is shown in Figure 1.

## Study instrument

A study instrument was developed which was face-validated, content-validated, and pilot-tested in the sample population. It included sections on informed consent, demographics, medical history, vaccination details, the effectiveness of the vaccine, and the safety profile of the vaccine. The participants of the study had the choice to choose to participate in the study by signing the consent form voluntarily. The demographics section of the study instrument contained information regarding age, gender, origin, nationality, occupation, income, and family details. Medical histories of participants were recorded including known allergies, co-morbidities, history of hospitalization, and history of COVID-19 infection. The vaccination details including type of vaccine, doses, and center of vaccination were inquired. Further, all questions regarding the safety of the vaccine in children including Adverse Drug Reactions and Side effects were logged. The severity of COVID-19 were classified according to NIH COVID-19 treatment guidelines as; A*symptomatic or pre-symptomatic infection, Moderate illness, Severe illness and* c*ritical illness* (15). The section was further divided into three sub-sections (a) observation of side effects after the first dose of the COVID-19 vaccine, (b) observation of side effects after the second dose of the COVID-19 vaccine and (c) observation of side effects after a booster dose of COVID-19 vaccine if available. This segment also recorded the severity, onset, and duration of commonly reported side effects in children.

## Validation of data collection form

Validation of the study instrument was done in two steps (a) face validation was done by two subject experts in the Medical and Pediatrics departments of tertiary care hospital, (b) content validation was done by an English language expert. Further, a pilot study was conducted in March 2022 with a representative sample of 60 participants. Any misleading questions were removed or modified and the study instrument was reviewed by the principal investigator of this study.

## Statistical analysis

Statistical analysis was done with SPSS version 25. The data was presented in tables, cross tabulations and frequency distributions for descriptive statistics. The correlation between various variables were estimated using the Chi Square test.

## Results

### Data collection

After applying inclusion and exclusion criteria, the data of 1,108 participants were included in the study (Figure 1). The majority of the children get vaccinated from vaccination centers (57.6%) followed by schools (31%). The classification of Participants included in the study based on their demographic indicators is shown in Table 1. The sample was demographically diverse with 544 (49%) male and 564 (51%) female children and adolescents. The median age of the participants was 16 years ranging from 12 to 18 years. All participants received the Pfizer vaccine which was approved by FDA for use in children and adolescents. Out of 1,108, the children partially vaccinated with the administration of only one dose were 801 (72.2%) while 307 (27.8%) were fully vaccinated with two doses.

**Table 1:** Gender, age, and vaccination status of participants.

	**Partially vaccinated (one dose)**	**Completely vaccinated (two doses)**	**Total**
**Gender**			
Male	416	129	545 (49%)
Female	385	178	563 (51%)
Total	801 (72.2%)	307 (27.7%)	1,108
**Age in years**			
12	64	31	95 (8.5%)
13	105	23	128 (11.5%)
14	114	37	151 (13.6%)
15	128	33	161 (14.5%)
16	130	46	176 (15.8%)
17	152	81	233 (21%)
18	108	56	164 (14.8%)
Total	801 (72.2%)	307 (27.7%)	1,108

Medical history of study participants demonstrates 27 (2.4%) children (16 male and 11 female) had a history of any known allergy while 26 (2.3%) children (12 male and 14 female) had a history of any co-morbid condition or a known disease including asthma, diabetes or cancer. Among all, 66 children (5.9%) had been hospitalized with any medical emergency in the last 2 years while the only child had a history of COVID-19 confirmed with PCR as shown in Table 2.

**Table 2:** Clinical features of study participants.

**Clinical feature**	**Response**	**Male**	**Female**	**Total**
History of allergy	Yes	16	11	27 (2.4%)
	No	529	552	1,081 (97.5%)
History of any disease	Yes	12	14	26 (2.3%)
	N0	533	549	1082 (97.6%)
History of hospitalization	Yes	47	19	66 (6%)
	No	498	544	1,042 (94%)
History of COVID (PCR confirmed)	Yes	0	1	1 (0.09%)
	No	545	562	1,107 (99.9%)

### Willingness to get the vaccine

**Figure 2:**
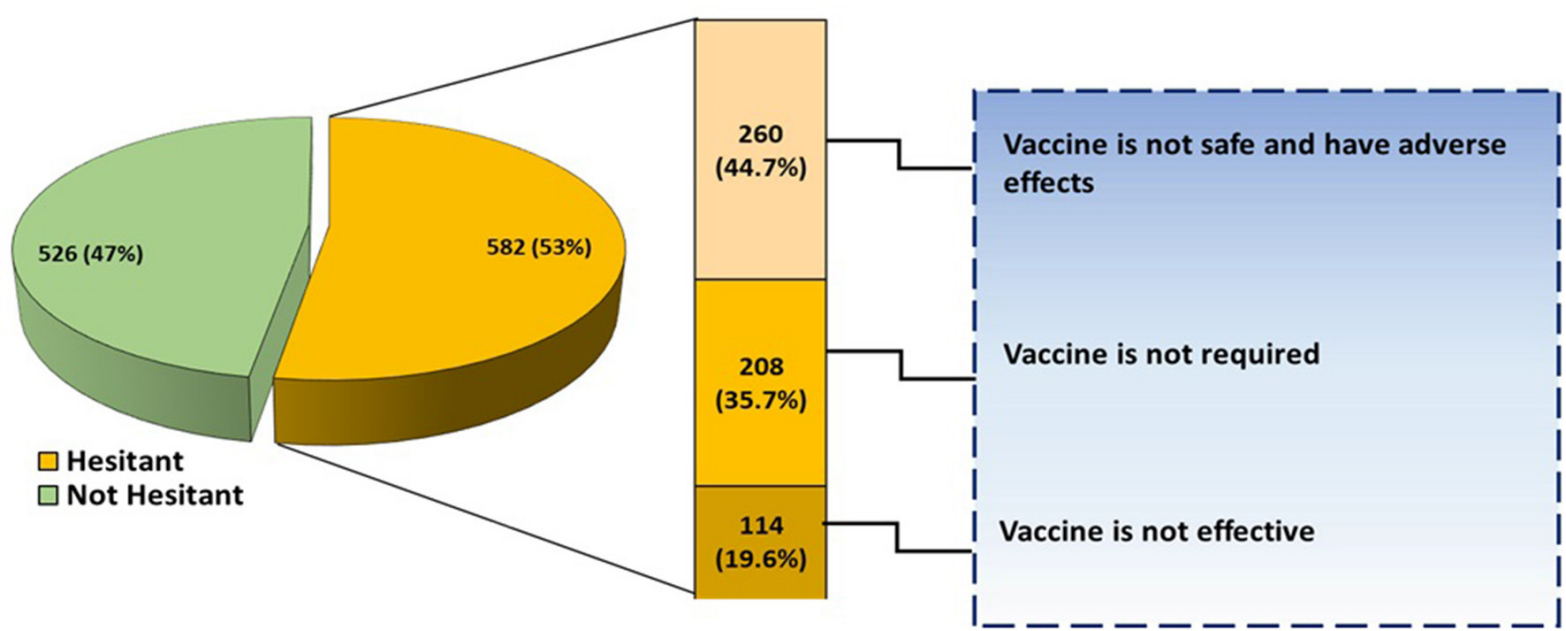
Willingness of participants to get the COVID-19 vaccine.

The willingness of the participant's parents/guardian in getting COVID-19 vaccination is shown in Figure 2. A total of 582 (53%) participants were hesitant about receiving COVID-19 vaccination while the remaining 526 (47%) were not afraid. Out of 582 participants who were hesitant about receiving COVID-19 vaccination, 260 (44.7%) assumed that the vaccine is not safe, 208 (35.7%) were of the view that the vaccine is not necessary while 114 (19.6%) doubted the effectiveness of COVID-19 vaccine (Figure 2).

Gender was very significantly associated (*p* = 0.003, CI = 5%) with the odds of receiving one or two doses of vaccine. Females were more likely to receive the 2nd dose that is full coverage of the vaccine as compared to male children of the same age (Table 1).

### Occurrence of side effects

The nature severity, onset, and duration of side effects are shown in Table 3. Out of 1,108 participants, 981 (88.5%) side effects were of mild nature (mostly pain at the site of injection), 118 (10.6%) were of moderate nature while only 9 (0.8%) participants experienced severe ADRs. However, no life-threatening ADR occurred in the studied population after the administration of the first or second dose of the COVID-19 vaccine. The onset of action of ADRs was 0–2 h in 277 (25%) participants, within 24 h in 757 (68.3%) participants, 2–3 days in 60 (5.4%) participants, 1 week in 13 (1.2%) while more than a week in only 1 (0.1%) participant. The reported ADRs lasted for 1 day in 240 (21.7%), 2–3 days in 672 (60.6%), 1 week in 173 (15.6%), and more than 1 week in 23 (2.1%) participants.

**Table 3:** Severity, onset, and duration of reported side effects.

	**Description**	**Frequency (number)**	**Percent (%)**
Severity	Mild	981	88.5
	Moderate	118	10.6
	Severe	9	0.8
The onset of side effects	0–2 h	277	25
	Within 24 h	757	68.3
	2–3 days	60	5.4
	1 week	13	1.2
	>1 week	1	0.1
Duration of side effects	1 day	240	21.7
	2–3 days	672	60.6
	1 week	173	15.6
	>1 week	23	2.1

**Figure 3:**
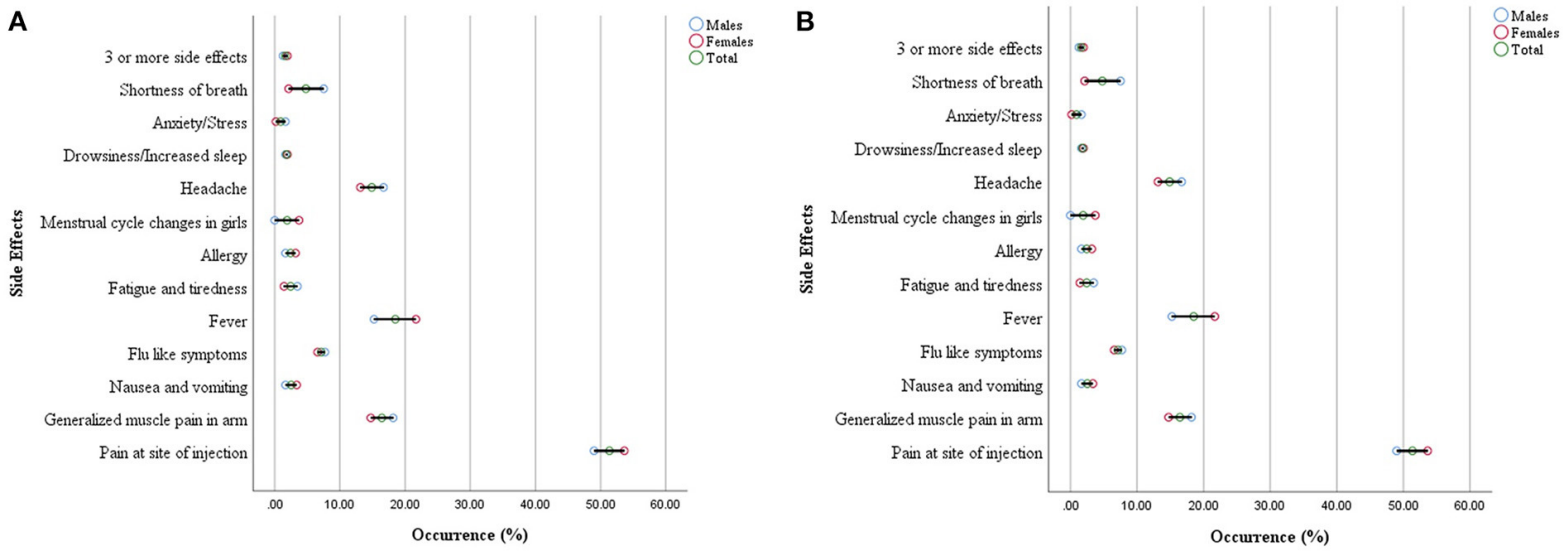
Gender distribution of reported side effects after the first dose **(A)** and second dose **(B)**.

**Figure 4:**
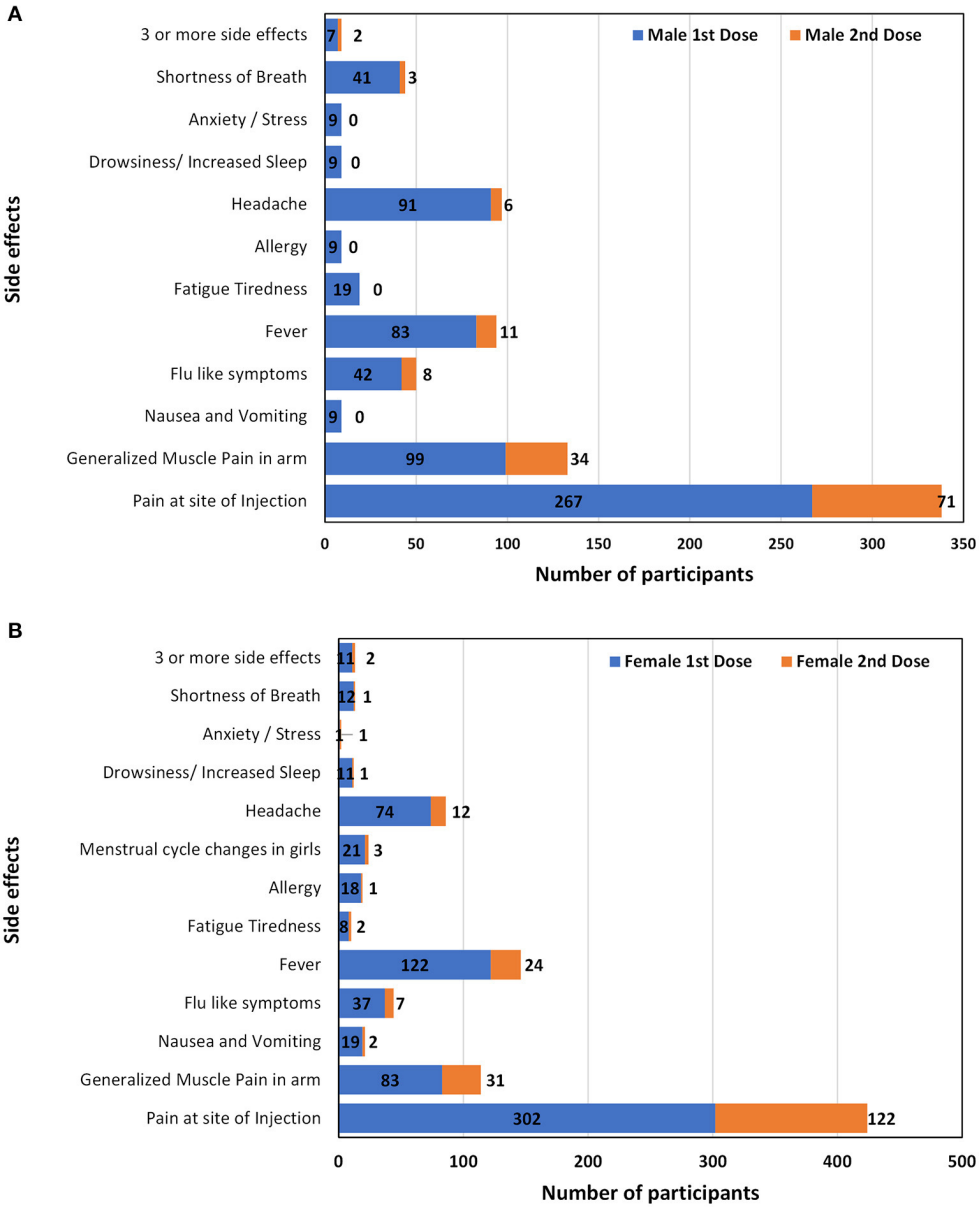
Comparison of side effects after administration of first and second dose in male **(A)** and female participants **(B)**.

**Figure 5:**
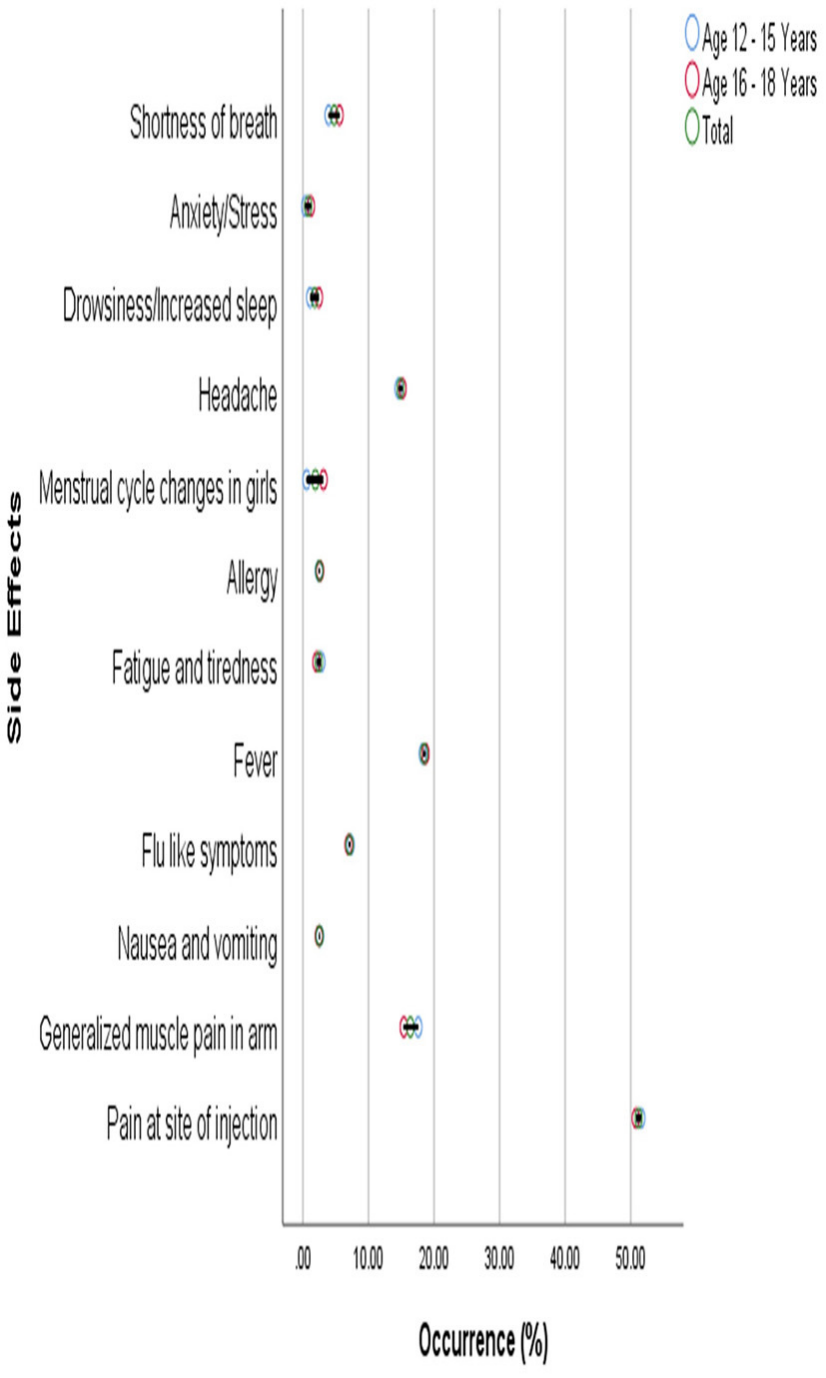
Age distribution of reported side effects after the first dose.

The commonly reported side effects in male and female participants after 1st dose of the vaccine are shown in Figure 3. The occurrence of side effects was significantly associated with gender (*P* = 0.00) when evaluated with Pearson's Chi-Square test as shown in Table 4. Pain at the site of injection, fever, Nausea, vomiting, and allergy were more commonly reported in female participants while generalized muscle pain, headache, shortness of breath, and fatigue/tiredness were more common in male participants. However, the side effects that appeared after 2nd dose were less as compared to 1st dose. The prevalence of side effects after 1st and 2nd doses in male and female participants is shown in Figure 4. The comparison of side effects in children aged 12–15 years and 16–18 years are shown in Figure 5. Pain at the injection site was reported in a higher number in younger children (12–15 years). While pain in combination with flu, headache, and shortness of breath as well as menstrual cycle changes are reported in higher numbers in adolescents (16–18 years). On the other hand, the age of children 12–18 years was not significantly associated (*p* = 0.07) with side effects after the 1st dose of the vaccine but had a very significant association (*p* = 0.008) after the 2nd dose of the vaccine.

**Table 4:** Association of gender with reported side effects (Pearson's Chi-Square test).

**Reported side effects**	**Male (*****n*** = **544)**		**Female (*****n*** = **564)**		**Total respondents (%)**	***P***
	***n***	**%**	***n***	**%**		
Pain at the site of injection	267	49.08	302	53.55	569 (51%)	0.00^*^
Generalized muscle pain in the arm	99	18.20	83	14.72	182 (16%)	
Nausea and vomiting	9	1.65	19	3.37	28 (2.5%)	
Flu-like symptoms	42	7.72	37	6.56	79 (7%)	
Fever	83	15.26	122	21.63	205 (18.5%)	
Fatigue tiredness	19	3.49	8	1.42	27 (2.4%)	
Allergy	9	1.65	18	3.19	27 (2.4%)	
Menstrual cycle changes in girls	0	0.00	21	3.72	21 (1.9%)	
Headache	91	16.73	74	13.12	165 (14.9%)	
Drowsiness/increased sleep	9	1.65	11	1.95	20 (1.8%)	
Anxiety/stress	9	1.65	1	0.18	10 (0.9%)	
Shortness of breath	41	7.54	12	2.13	53 (4.8%)	
Three or more side effects	7	1.29	11	1.95	18 (1.6%)	

* Significant.

### Effectiveness of vaccine

The effectiveness of the COVID-19 vaccine was evaluated by the reoccurrence of COVID-19 infection in vaccinated children and it was observed that 99.9% of the participants did not develop an infection when evaluated through PCR test.

## Discussion

The purpose of the study was to evaluate the positive and negative outcomes of the vaccine in Children in Pakistan following COVID-19 vaccination. The major vaccinated group among children is 12–18 years in Pakistan due to the approval of vaccination only in this age group. This is in contrast to the international picture where FDA has approved vaccination for CA as young as 4 months up to 18 years. The findings indicate that majority (99.9%) of study participants got the Pfizer COVID vaccine shot. The number is likely to relate more to vaccine availability at the time of the study in vaccination than personal choice.

Among study participants, 72.2% were partially vaccinated with one dose while 27.7% were fully vaccinated with two doses. In this study, children 12–18 years who received one or both doses Pfizer vaccine experienced a reduced risk of COVID infection, which is precisely the prevention of infection in 99.9% of cases. This finding is in line with prior research that a person fully vaccinated with two doses of Pfizer acquired 95% protection against serious COVID disease in 16 years and older respondents. Moreover, the effectiveness of 90–100% was observed in population subgroups defined based on gender, age, and ethnicity (11). The ratio of females was higher who received two doses (i.e., 57.9%) compared to males (i.e., 42%). In previous studies, two-dose regimens of the COVID-19 vaccine improved protective efficacy and immunity against COVID-19 (16).

A good safety profile of COVID vaccination in children with mild and transient side effects was observed. The majority reported side effects to be mild (88.5%) with an onset of 24 h (68%) and a duration of 2–3 days (60.6%). This finding is similar to previous findings where vaccination is dominated by local symptoms (5). We confirmed the findings of prior research from other populations describing the low incidence of mild, transient, and self-limiting side effects. The incidence of Adverse Drug Reactions (ADRs) is very low (0.45%) contributing to a good safety profile in children.

The hesitancy to receive COVID-19 vaccination was agreed by 52% of Children while the remaining 48% reported no fear or hesitancy to receiving vaccine dose. The most common reason for fear and hesitancy to receive a vaccine was an assumption by respondents that the vaccine has side effects and is not safe enough. This is in line with emergency use authorizations of COVID-19 vaccines by FDA internationally and numerous studies showed concern of the masses for vaccine safety.

The results expressed the incidence of local and systemic side effects of the vaccine in CA of mild and moderate intensity. Some common side effects reported are pain at the site of injection, muscle pain, headache, and fever. These findings correlate with most of the findings from clinical trials and studies conducted in adults (17, 18).

The results of the study depict gender significantly influencing the incidence of vaccine side effects (*p* = 0.00, CI = 5%). More prominently, females reported a higher incidence of pain at the site of injection (53.5%), nausea (3.37%), allergy (3.2%), and menstrual cycle changes (3.72%). Further, the menstrual cycle changes in adolescent girls have also been recorded to be (1.9%) which is also parallel to the findings from other studies which reported COVID infection and COVID vaccination especially mRNA vaccines can make changes in the menstrual cycle (19).

On the other hand, male children reported a higher incidence of generalized muscle pain (18.2%), fatigue (3.5%), anxiety (1.65%), and shortness of breath (7.54%). However, adolescent boys reported shortness of breath alone (0.8%) or in combination with fever (3.9%). The incidence of this effect is much lower in females as compared to adolescent males. Shortness of breath may or may not be attributed to the long-term development of pericarditis and myocarditis. Similar findings from other research studies have also been observed. Myocarditis was already documented as a rare complication of Coronavirus mRNA vaccines predominantly in adolescent males. The incidence is low and still outweighs the benefit in a risk-benefit ratio of vaccination (20).

The side effects of the second dose of the vaccine are similar to the first dose. Gender (*p* = 0.003) and age (*p* = 0.00) are significantly correlated to the incidence of side effects after 2nd dose. 91% of children reported severity of symptoms was mild, onset 24 h (70%), and duration 2–3 days (59%).

The findings of the study have various viewpoints. Vaccination of children and adolescents expressed many direct and indirect benefits of preventing disease and halting community transmission. Although, children had a less symptomatic disease but played a significant role in the transmission of disease highlighted in many research studies globally. Given the observed efficacy of the COVID-19 vaccine in children, it is inferred that vaccination will help prevent asymptomatic infection in adolescents and children, thereby broadening community protection.

## Conclusions

The study documented some commonly reported side effects in Children and Adolescents including local and systemic both, therefore making them predictable. The most common reasons for hesitance were the assumption that the vaccine is not safe.

COVID-19 vaccine was well tolerated by children with good safety profile and very less adverse drug reactions. The reported side effects of the vaccine have a significant correlation with some demographic factors such as the Age and Gender of Children. The reported side effects were mainly transient mild to moderate and appeared with an onset of an average of 24 h and a duration of 2–3 days. Gender is significantly associated with reported side effects. Females reported a higher incidence of pain at the site of injection, nausea, allergy, and menstrual cycle changes as compared to their male counterparts. Male children reported a higher incidence of generalized muscle pain, fatigue, anxiety, and shortness of breath.

## Limitations and recommendations

Though the study successfully highlighted the most common side effects of COVID vaccination in CA in Pakistan which were not known, it has some limitations. First, the research study was conducted in a specific area, i.e., the province of Punjab with an inflow of residents from all other provinces and surrounding cities. Second, the research data was collected with a convenience sampling method. Third, the data was collected a few months after the vaccination process started in children in Pakistan, hence the recall bias may have hindered in reporting of vaccine outcomes accurately by children. These limitations may influence the generalizability of the current study results to the entire population of Pakistan.

The way forward includes the design and implementation of new seroprevalence studies focused on vaccine effectiveness and safety profile under 12 years Children. Also, the inclusion of key stakeholders including health ministry, policymakers, and practitioners may be considered to implement educational plans for parents and children. Targeted counseling programs before and after vaccination might be initiated to help overcome potential post-vaccination side effects in the context of demographic variations.

## Data availability statement

The raw data supporting the conclusions of this article will be made available by the authors, without undue reservation.

## Ethics statement

The studies involving human participants were reviewed and approved by Office of Research Innovation and Commercialization (ORIC), Lahore College for Women University (LCWU), Lahore. Written informed consent to participate in this study was provided by the participants' legal guardian/next of kin.

## Author contributions

ZZ has collected the data, compiled the data, and made the initial draft of manuscript. MU analyzed the data and drafted the manuscript. KF and AA collected the data. HK conceived the idea, supervised the study, and finalized the manuscript. RB revised the manuscript and contributed in proof reading. All authors contributed to the article and approved the submitted version.
